# Enzymatic Blockade of the Ubiquitin-Proteasome Pathway

**DOI:** 10.1371/journal.pbio.1000605

**Published:** 2011-03-29

**Authors:** Robert Ernst, Jasper H. L. Claessen, Britta Mueller, Sumana Sanyal, Eric Spooner, Annemarthe G. van der Veen, Oktay Kirak, Christian D. Schlieker, Wilhelm A. Weihofen, Hidde L. Ploegh

**Affiliations:** 1 Whitehead Institute for Biomedical Research, Cambridge, Massachusetts, United States of America; 2 Department of Molecular and Cellular Biology, Harvard University, Cambridge, Massachusetts, United States of America; 3 Department of Biology, Massachusetts Institute of Technology, Cambridge, Massachusetts, United States of America; National Cancer Institute-NIH, United States of America

## Abstract

Ubiquitin-dependent processes control much of cellular physiology. We show that expression of a highly active, Epstein-Barr virus-derived deubiquitylating enzyme (EBV-DUB) blocks proteasomal degradation of cytosolic and ER-derived proteins by preemptive removal of ubiquitin from proteasome substrates, a treatment less toxic than the use of proteasome inhibitors. Recognition of misfolded proteins in the ER lumen, their dislocation to the cytosol, and degradation are usually tightly coupled but can be uncoupled by the EBV-DUB: a misfolded glycoprotein that originates in the ER accumulates in association with cytosolic chaperones as a deglycosylated intermediate. Our data underscore the necessity of a DUB activity for completion of the dislocation reaction and provide a new means of inhibition of proteasomal proteolysis with reduced cytotoxicity.

## Abstract 2

A viral ubiquitin-specific protease domain blocks cellular protein turnover by preemptive removal of ubiquitin chains and establishes an essential role for deubiquitylation in the removal of unfolded proteins from the endoplasmic reticulum.

## Author Summary

Constant turnover of proteins is part of normal cellular physiology. Newly synthesized proteins that fail to fold are recognized by dedicated receptors and tagged for immediate degradation. The tag usually consists of a chain of a small protein, ubiquitin, and is recognized by the proteasome. We introduce a new tool to interfere with proteasomal degradation: a ubiquitin-specific protease domain (EBV-DUB) derived from Epstein-Barr Virus. This EBV-DUB, when expressed in mammalian cells, preemptively removes ubiquitin chains (deubiquitylation) and so frustrates substrate recognition and engagement by the proteasome. The natural history of misfolded, secretory proteins is poorly understood because of the strict coupling of recognition, tagging for degradation, and proteolysis. The EBV-DUB uncouples these processes and stabilizes short-lived intermediates, an activity that helped us to address the question of how such misfolded proteins are extracted from the relevant cellular compartments. Our data are consistent with the idea that unfolded substrates targeted for degradation are threaded through a narrow pore of the chaperone protein known as p97. In order to pass through the pore, the protein must first have any already attached ubiquitin chains removed; a second cycle of ubiquitylation is then required to allow engagement of the proteasome. Entry of substrate into the proteolytic chamber again requires removal of ubiquitin. We thus propose two rounds of ubiquitin attachment and removal in the course of the extraction and degradation of misfolded proteins.

## Introduction

Protein quality control and ubiquitin-dependent degradation are essential for cellular homeostasis and survival [1]. The ubiquitin-proteasome-system (UPS) is responsible for the turnover of most cytosolic proteins. Likewise, secreted and membrane proteins that do not fold properly or fail to associate with their requisite partners in the ER are delivered to the cytosol and then destroyed by the UPS [2]. To facilitate this reaction, one or several dedicated receptors recognize misfolded ER-luminal proteins, which are then recruited to the dislocation machinery and rendered accessible to the cytosolic ubiquitylation apparatus. For both cytosolic and ER-derived substrates, attachment of polyubiquitin (poly-Ub) chains by an enzymatic E1-E2-E3 cascade is the signal for proteasomal degradation [3]. Poly-Ub chains serve as a recognition signal also for protein dislocation from the ER [4]. When an ER-derived misfolded protein gains access to the cytosol, the attachment of a poly-Ub chain recruits the cytosolic ATPase p97/VCP/CDC48 (Swiss-Prot ID: P55072) and its associated co-factors Ufd1-Npl4 [5]–[7], believed to provide the force required for extraction of substrate from the ER. It is not known whether these Ub-chains are utilized as a handle to exert a mechanical force or whether they target the dislocated protein directly to the proteasome [5],[6],[8].

The 19S lid of the 26S proteasome and p97/VCP/CDC48 both occur in association with ubiquitin ligase and deubiquitylating activities [9],[10]. Ubiquitylation is a dynamic process, tightly controlled by a collection of associated ubiquitin-processing factors, both at the level of the proteasome and at the level of p97 [9],[10]. Ubiquitylation and its reverse reaction, catalyzed by deubiquitylating enzymes (DUBs), are crucial for p97-mediated dislocation and for proteasome function [3],[5]. Impairment of p97-associated DUB activity can block substrate dislocation [11],[12]. The removal of poly-Ub chains by DUBs associated with the proteasomal lid precedes the threading of unfolded proteins through a narrow pore into the proteolytic chamber of the core 20S proteasome [1],[13],[14]. The removal of Ub prior to degradation also recycles this essential modifier and replenishes the cellular pool of free Ub. It follows that DUB activity can have distinct outcomes for proteasomal turnover of proteins: some DUBs facilitate degradation, whereas others may stabilize proteins destined for degradation.

Removal of glycoproteins from the ER involves multiple distinct enzymatic reactions: ubiquitylation, deubiquitylation, deglycosylation, and ATP-dependent dislocation [2]. How are the opposing activities of ubiquitylation and debuiquitylation coupled in the course of extraction from the ER and delivery to the proteasome? The activity of DUBs is no less carefully controlled than that of the ligases that carry out ubiquitylation. The catalytic domains of DUBs, both cellular and viral, are flanked by often sizable segments that likely mediate such control [12],[15],[16]. We reasoned that the expression of a highly active DUB protease domain, excised from its normal context, might preemptively remove Ub from substrates targeted for degradation and stabilize them. We chose the protease domain of the Epstein-Barr Virus (EBV) large tegument protein (BPLF1, Swiss-Prot ID: P03186) for that purpose. We use this EBV-DUB to cause an enzymatic blockade of the UPS and show that its expression uncouples dislocation from degradation. Our data demonstrate that protein dislocation from the ER requires a DUB activity upstream of p97-mediated extraction from the ER. Furthermore, a side-by-side comparison of different experimental strategies that impede degradation of a misfolded ER-luminal substrate enabled us to identify this substrate's interactors at distinct stages en route to degradation. This allows us to propose a timeline for the discrete steps involved.

We further identify the ER-luminal machinery for disulfide bridge formation as a putative target of eeyarestatin-I, a small-molecule inhibitor of dislocation. The cytosolic co-chaperone recruiter BAT3 (Swiss-Prot ID: P46379) surfaces as a specific interactor of an ER-derived—and now cytosolic—substrate when the UPS is blocked by the EBV-DUB. Our data suggest a previously unanticipated function of cytosolic chaperones, namely to cope with ER-derived misfolded proteins that arrive in the cytosol. The consequences of EBV-DUB expression are less toxic than those caused by pharmacological proteasome inhibitors and might find wide application in cell biology.

## Results

### EBV-DUB Is a Highly Active, Viral Ubiquitin-Specific Protease

**Figure 1:**
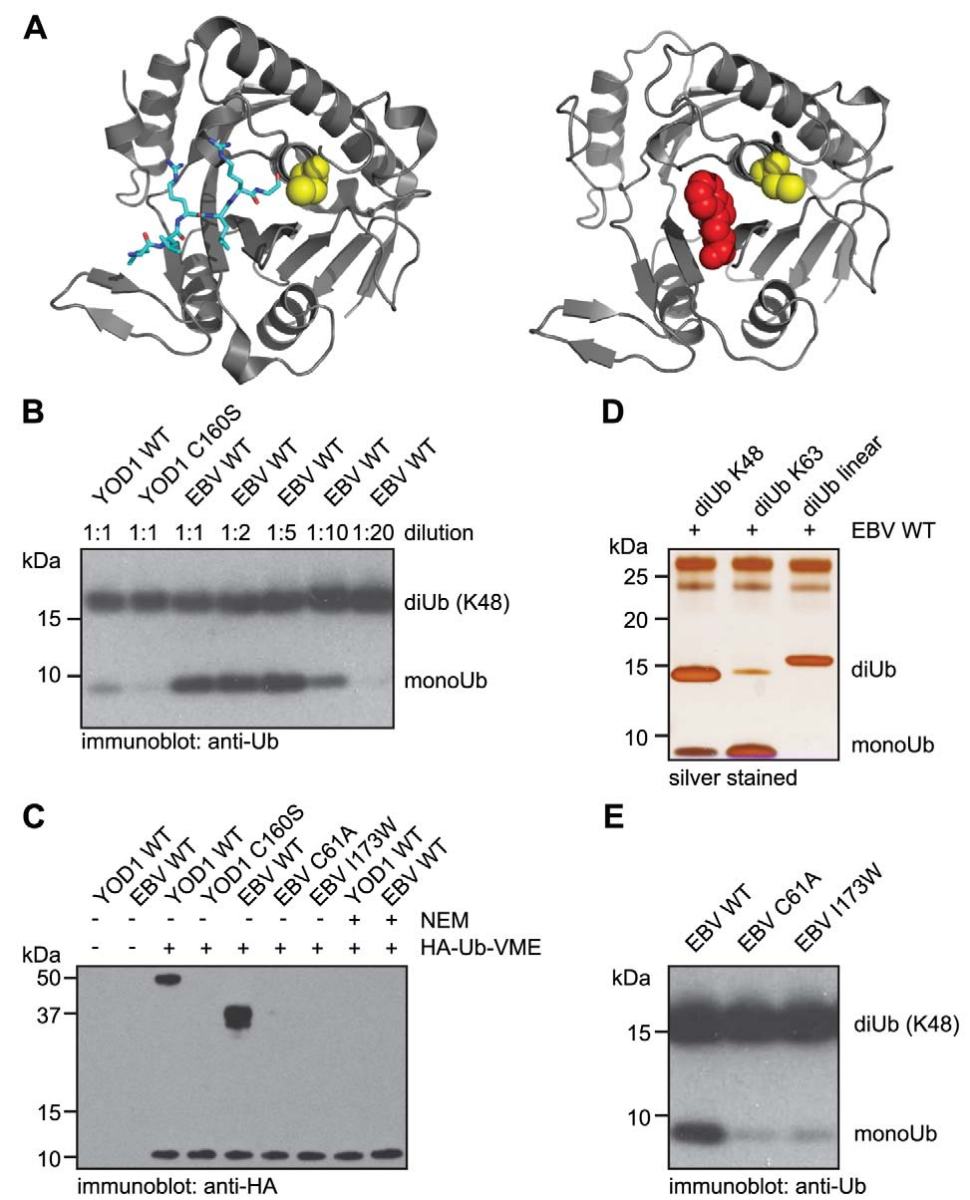
EBV-DUB is a highly active enzyme in vitro. (A) The left panel shows a ribbon representation of the M48 protease domain from the mouse cytomegalovirus (MCMV) (PDB code: 2J7Q) [16]. Depicted in cyan is the extended C-terminus of Ub in the M48 active-side cleft pointing towards the catalytic cysteine (C23), shown in yellow. The right panel shows a structural model of EBV-DUB I173W, with the catalytic cysteine (C61) depicted in yellow and the tryptophane 173 point mutation, blocking the catalytic cleft, shown in red. (B) YOD1 WT, YOD1 C160S, and EBV-DUB WT (1 µg) were incubated with 0.5 µg of K_48_-linked diUb for 3 h at 37°C. The amount and concentration of the EBV-DUB WT was titrated as indicated. The reaction was stopped by boiling in sample buffer, subjected to SDS-PAGE, and immunoblotted with anti-Ub antibody. (C) Purified YOD1 and EBV-DUB (WT and indicated mutants) were incubated at a molar ratio of 1∶1 with HA-Ub-VME, an activity-based probe [17], and subjected to SDS-PAGE. When indicated, NEM was included in the reaction mixture. HA-Ub-VME adduct formation was visualized by immunoblotting with anti-HA antibodies. Purified EBV-DUB is proteolytically processed and runs as a double band, however both forms promote adduct formation with the HA-Ub-VME probe. (D) K_48_-linked, K_63_-linked, or linear diUb (0.5 µg) was incubated for 3 h at 37°C in a total volume of 10 µl with EBV-DUB WT (1 µg). The reaction mixture was subjected to SDS-PAGE and silverstained. (E) EBV-DUB WT, the C61A mutant, and the I173W mutant of the EBV-DUB were incubated with K_48_-linked diUb (0.5 µg) for 3 h at 37°C. The reaction mixture was subjected to SDS-PAGE and immunoblotted with anti-Ub antibody.

We aimed to shift the balance of ubiquitylation towards deubiquitylation through enforced expression of a highly active DUB. To this end we employed the protease domain (aa1-270) of the EBV BPLF1 gene (Figure 1A) [16]. The isolated protease domain (EBV-DUB) hydrolyzed K_48_ Ub-linkages more readily (>10-fold) than preparations of the cellular DUB YOD1 (Swiss-Prot ID: Q5VVQ6) (Figure 1B). The EBV-DUB was an excellent substrate for the activity-based probe HA-Ub-VME [17], but not when the putative catalytic cysteine was substituted to alanine (C61A) or when Ub-binding was abolished through blocking the catalytic cleft of the EBV-DUB by an I173W mutation (Figure 1A,C). BPFL1 is active towards both K_48_- and K_63_-linked di-Ub, as well as NEDD8 [18], but not against linear di-Ub despite its topological similarity to K_63_-linked Ub (Figure 1D) [19]. The cellular function of BPLF1's DUB activity remains largely unknown [20]. Our experiments do not address the hydrolysis of poly-Ub chains of other linkage types, including K_11_, or chains of mixed topologies, all of which could contribute to proteasomal targeting to different degrees [21]. K_63_ linkages have been linked primarily to endocytosis and other non-proteasomal events [22] but could contribute to protein homeostasis as well [23],[24]. As a control for all subsequent experiments, we employed the I173W mutant unable to bind and hydrolyze Ub-chains (Figure 1A,C,E).

**Figure 2:**
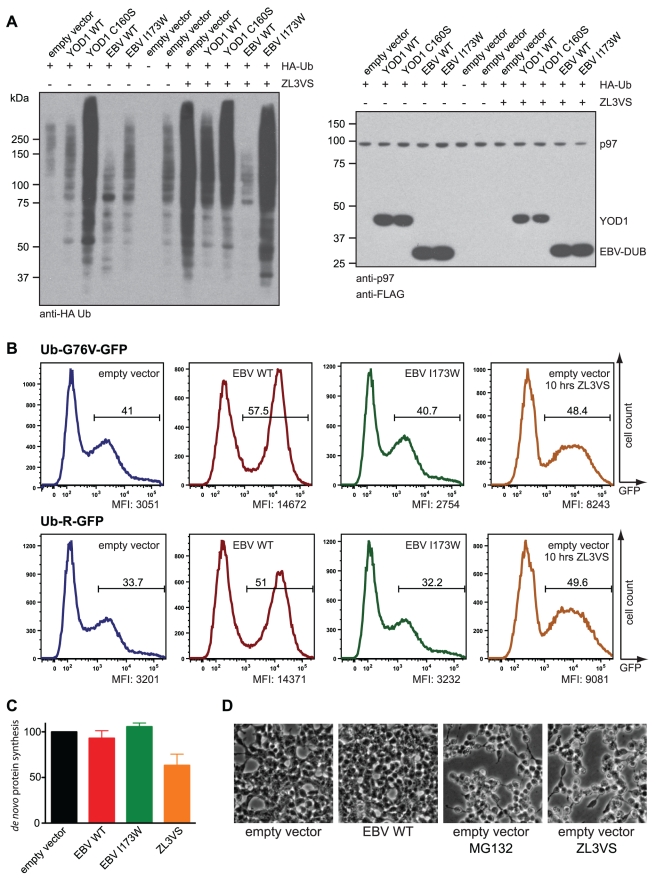
A viral DUB switches the cellular ubiquitylation balance towards deubiquitinylation and blocks proteasomal degradation. (A) Lysates of 293T cells transfected as indicated and immunoblotted with anti-HA, anti-p97, and anti-Flag antibodies. Where indicated, cells were treated with for 10 h with 10 µM ZL3VS. (B) Flow-cytometric analysis of 293T cells treated and co-transfected with as indicated. The gate was set to identify GFP-positive, live cells. Quantified is the median fluorescence intensity (MFI) of GFP-positive cells. (C) Pulse-labeling experiment and quantification of *de novo* protein synthesis. Where indicated, cells were starved and radiolabeled in the presence of 50 µM ZL3VS. Radioactivity incorporated by control cells was normalized to 100%. The error bars depict the standard deviation (*n* = 4). (D) Morphology changes of 293T cells 26 h after transfection and after treatment for 20 h with 10 µM of indicated proteasome inhibitors.

Expression of a FLAG tagged variant of the wild-type EBV-DUB in 293T cells resulted in a substantial downward shift of polyubiquitylation in HA-ubiquitin expressing cells (Figure 2A). In contrast, the expression of a cellular, less active DUB (YOD1 WT) failed to do so. Consistent with previous observations, the catalytically inactive mutant (YOD1 C160S) caused accumulation of polyubiquitylated proteins, presumably due to stalled dislocation [11]. The efficiency with which the viral DUB eliminated polyubiquitylated conjugates in living cells is even more apparent when the activity of the proteasome is blocked by prior exposure of cells to ZL3VS: polyubiquitylated proteins now accumulated in control cells but were largely absent from EBV-DUB WT cells when examined at similar sensitivity of detection (Figure 2A). To corroborate our findings, we repeated our experiments in the absence of co-transfected HA-Ub. Immunoblots using antibodies directed against ubiquitin or specific for Lys48-linked ubiquitin revealed diminished polyubiquitylation in EBV-DUB WT cells, but not in control cells (Figure S1).

A strong reduction in polyubiquitylation should affect protein turnover globally. Therefore we analyzed the effect of EBV-DUB expression on steady-state levels of two short-lived, cytosolic GFP variants: the ubiquitin-fusion degradation (UFD) substrate Ub-G76V-GFP and the N-end rule substrate Ub-R-GFP (Figure 2B) [25]. Both proteins are unstable and their detection improves upon inhibition of the UPS. Ub-R-GFP is processed by cellular ubiquitin hydrolases, which results in exposure of arginine as the N-terminal destabilizing residue [26]. Ub-G76V-GFP cannot be processed by cellular hydrolases, but the fusion with ubiquitin itself serves as a degradation signal [27].

Co-expression of Ub-G76V-GFP and EBV-DUB WT gave rise to a population with high GFP fluorescence, best illustrated by the 5.3-fold higher median fluorescence intensity of GFP-positive cells (MFI) as compared to control cells and the 1.8-fold increased MFI compared to cells treated with the proteasome inhibitor ZL3VS (Figure 2B). Similarly, Ub-R-GFP and EBV-DUB co-expressing cells exhibited a 4.4-fold higher MFI compared to control cells and a 1.6-fold higher MFI compared to ZL3VS-treated cells, apparent also when titrating ZL3VS (Figure S2). As expected, the relative fraction of GFP-positive cells increased when protein degradation was impaired (Figure 2B). ZL3VS is an efficient inhibitor of the chymotryptic and the peptidyl-glutamyl peptide hydrolyzing activities of the proteasome and impairs its tryptic activity by ∼50% [25],[28]. Taking this into account, our flow cytometric data on two well-established, short-lived proteins suggest a near complete blockade of the UPS [25],[29]. Both the N-end rule and UFD pathway are affected, likely at an ubiquitinyl-dependent step in commitment of the substrate to the proteasome.

Ubiquitylation and protein turnover are central to many cellular processes. Overexpression of a highly active DUB or proteasomal inhibition by small molecules might affect the physiology of a cell in many ways. Pharmacological inhibition of the proteasome impairs *de novo* protein synthesis [30],[31]. In our hands, treatment of cells with ZL3VS for 40 min resulted in ∼40% reduced incorporation of radioactivity in a 10 min pulse labeling experiment when compared to control and EBV-DUB expressing cells (Figure 2C). Thus, *de novo* protein synthesis was impaired upon pharmacological inhibition but remained unperturbed in EBV-DUB cells. Prolonged treatment (20 h) of cells with proteasome inhibitors caused growth arrest and changes in cell morphology, consistent with the known ability of proteasome inhibitors to induce apoptosis and cell cycle arrest [25],[32]. EBV-DUB cells appeared normal at times of cultivation where ZL3VS treated cells were morphologically aberrant (>26 h) (Figure 2D), but after much longer cultivation they, too, succumbed, presumably because the continued operation of the UPS is essential for cell survival.

### Expression of EBV-DUB Blocks Protein Turnover

**Figure 3:**
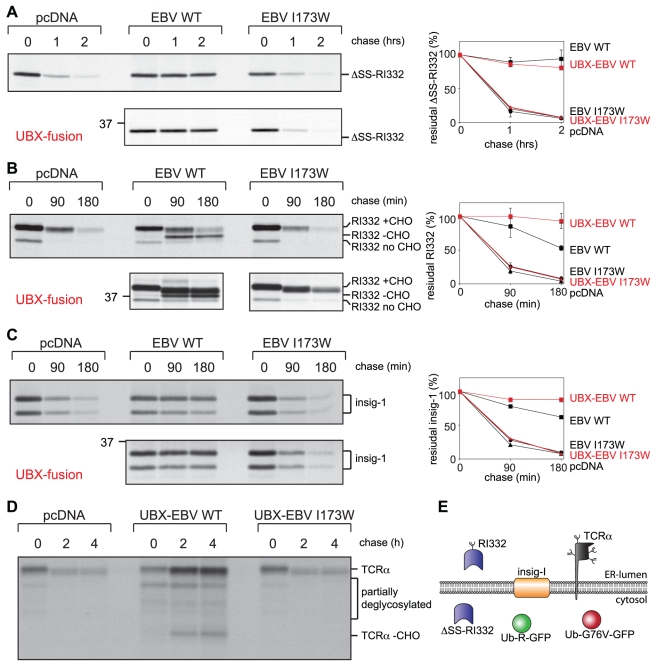
Expression of a viral DUB blocks degradation of cytosolic, ER-luminal, and ER-membrane proteins. (A–D) Pulse-chase analysis with unstable model substrates: (A) cytosolically localized ΔSS-RI_332_, (B) the ER-luminal glycoprotein RI_332_, (C) Insig-1, a multi-spanning membrane protein of the ER, and (D) TCRα, a glycosylated protein with one transmembrane helix. (E) Unstable model proteins used throughout this study are shown schematically. After indicated chase times, the model substrates were immunoprecipitated, subjected to SDS-PAGE, and quantified (right panels). The influence of targeting the EBV-DUB to p97 through installation of an UBX-domain (red symbols) versus non-targeted EBV-DUB (black symbols) is tested by direct comparison of model substrate stabilization. The error bars correspond to the standard deviation (*n* = 3). See Figure S3 for UBX-mediated targeting to p97.

Does expression of the viral DUB affect protein degradation directly? To allow a direct comparison of an ER-derived and cytosolic substrate, we employed RI_332_, a C-terminally truncated form of ribophorin-I that is commonly used as a model to study dislocation and degradation of ER-luminal proteins [11],[33],[34]. When devoid of its N-terminal signal sequence (ΔSS-RI_332_) the protein fails to enter the ER, cannot be glycosylated, and remains cytosolic, but is otherwise identical to RI_332_ (see schematic representation in Figure 3E). The cytosolic UPS substrate ΔSS-RI_332_ is turned over in both control and EBV-DUB I173W cells (t_1/2_ = 20 min), but its degradation was blocked by expression of EBV-DUB WT (Figure 3A). This confirms our flow cytometric data on Ub-R-GFP and Ub-G76V-GFP (Figure 2B) and demonstrates an efficient blockade of the UPS imposed by EBV-DUB WT. An arrest of the UPS should also affect the degradation of ER-derived proteins. When equipped with its natural signal sequence, RI_332_ is translocated into the ER and glycosylated but rapidly destroyed in control and EBV-DUB I173W cells (t_1/2_ = 44 min) (Figure 3B) [33].

The banding pattern observed in these experiments requires explanation. The lower band at the 0 min chase time point corresponds to ER-luminal, non-glycosylated RI_332_ (RI332 no CHO) with its signal sequence removed, while the upper band is the glycosylated form of RI_332_ (RI332 +CHO) [11]. Removal of the *N*-linked glycan by cytosolic *N*-glycanase converts asparagine at the site of glycan attachment to aspartate (N275D) [35]. As a consequence, deglycosylated RI_332_ (RI332-CHO) shows altered electrophoretic mobility. This form was readily apparent in EBV-DUB WT cells after a 90 min chase and beyond (Figures 3B, S3) and can arise only as a consequence of glycan removal from previously glycosylated RI_332_. Such deglycosylated intermediates are normally rapidly degraded by the proteasome and therefore escape detection, unless the activity of the proteasome is compromised [33].

Co-expression of the EBV-DUB stabilized RI_332_, but did not do so completely (Figure 3B). Since dislocation and proteolysis are at least to some extent coupled processes [33],[36], we reasoned that some Ub-chains present on ER-derived dislocation substrates might not be accessible to the EBV-DUB. We therefore targeted the viral DUB domain to p97, the “motor of dislocation,” by equipping it with the UBX domain of YOD1 to copy the strategy employed by this cellular DUB. This chimeric protein (UBX-EBV WT) associated with p97 (Figure S4) and blocked degradation of the ER-derived RI_332_ substrate completely (Figure 3B, UBX-fusion).

The degradation of two unrelated membrane proteins—the polytopic transmembrane protein insig-1 and the glycosylated α-chain of the T-cell receptor (TCRα) with one transmembrane helix (Figure 3E)—were likewise affected. Expression of UBX-EBV WT halted the turnover of myc-tagged insig-1, an ER-localized transmembrane protein that regulates cholesterol synthesis (Figure 3C) [8]. Of note, insig-1-myc is not a glycoprotein but has two alternative start codons yielding two translation products with distinct electrophoretic mobilities [37]. Also the degradation TCRα, an unstable protein when expressed in the absence of other T-cell receptor subunits, was blocked upon co-expression of UBX-EBV WT (Figure 3D). In summary, the EBV-DUB arrests turnover of cytosolic and ER-derived proteins. In all cases, targeting of the viral DUB to p97 improved the stabilization of ER-derived substrates, but its activity towards cytosolic substrates of the UPS persisted (Figures 3, S5).

### Uncoupling Protein Dislocation and Degradation

**Figure 4:**
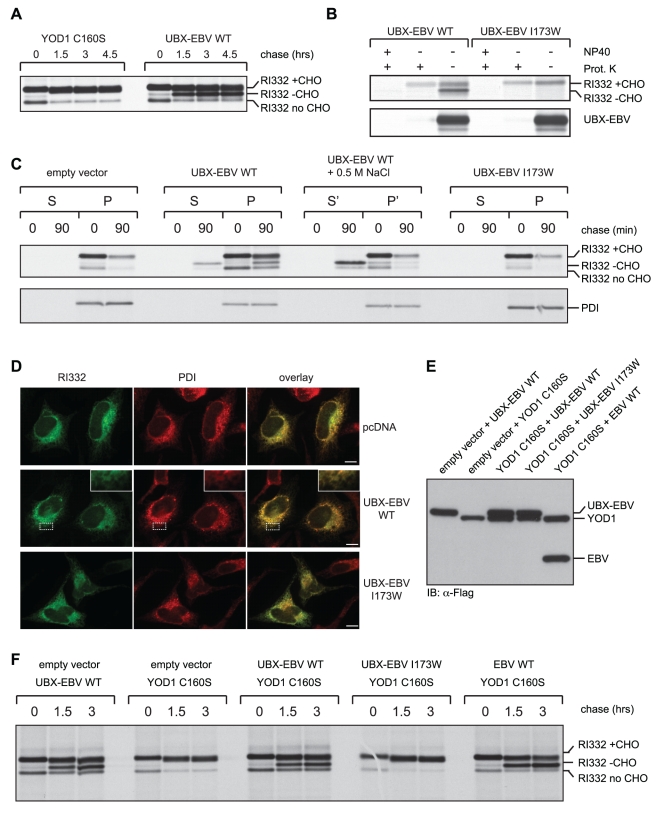
ER-dislocation and protein degradation are uncoupled by p97-targeted EBV-DUB. (A) 293T cells were co-transfected with RI_332_ and the indicated constructs and subjected to pulse chase analysis. (B) 293T cells were co-transfected with the indicated constructs, disrupted mechanically, and treated with proteinase K and/or NP40 as indicated. The UBX-EBV-DUB served as control for a cytosolic protein. (C) 293T cells were co-transfected as indicated and pulse labeled. After 0 min or 90 min of chase, the cells were subjected to a subcellular fractionation experiment, after selective permeabilization of the plasma membrane with Perfringolysin O. RI_332_ and PDI were retrieved after fractionation via immunoprecipitation. When shown (S'/P'), the fractionation was performed in the presence of high salt (0.5 M). (D) Immunofluorescence of HeLa cells co-transfected with RI_332_ and the indicated constructs. Endogenous PDI (red) served as an ER-marker and RI_332_ (green) was detected via the HA-epitope tag. Scale bar  =  10 µm. (E) 293T cells were co-transfected with indicated YOD1, EBV-DUB, and RI_332_ constructs. Expression levels of YOD1 and the EBV-DUB were determined from SDS-lysates after immunoblotting against the N-terminal Flag-epitope. (F) A pulse chase experiment was performed with 293T transfected as in Figure 4E. The block in dislocation induced by YOD1 C160S can be rescued by co-expression of UBX-EBV WT and EBV-WT.

The occurrence of the deglycosylated RI_332_ intermediate that accumulated in UBX-EBV WT and in EBV WT cells (RI_332_ –CHO; Figure 3B) was informative. Since *N*-glycanase is confined to the cytosol [35],[38], this observation immediately suggested that the single *N*-linked glycan of RI_332_ gained cytosolic exposure and therefore that dislocation must have occurred, entirely or in part. However, when turnover of RI_332_ was inhibited by expression of YOD1 C160S, the deglycosylated form RI_332_ was not observed, even after long chase periods (Figure 4A), indicating its confinement to the ER. The appearance of deglycosylated intermediates was not specific for the ER-luminal RI_332_ but was readily observable as well for TCRα when co-expressed with UBX-EBV WT (Figure 3D). For reasons that remain to be determined, we consistently observe greater recovery of label for TCRα at later chase points. It is possible that detergent extraction of newly synthesized TCRα is somehow less efficient than material that has left the site of membrane insertion. We do not observe a similar discrepancy for the other substrates analyzed, insig-1 and RI_332_.

We confirmed cytosolic accessibility of the deglycosylated RI_332_ intermediate by a proteinase K protection experiment in mechanically disrupted cells (Figure 4B). In the absence of detergent, only deglycosylated RI_332_ (RI332 – CHO) was accessible to protease. The glycosylated, ER-luminal form (RI332 + CHO) was not affected by the protease under identical conditions and serves as an internal control for membrane integrity (Figure 4B). Proteinase K sensitivity of deglycosylated RI_332_ implied that a substantial portion of RI_332_, if not RI_332_ in its entirety, was exposed to the cytosol in UBX-EBV WT expressing cells. To further corroborate this result, we performed a fractionation experiment in which we made use of the pore-forming toxin Perfringlolysin O (PFO) [39]. UBX-EBV WT cells were pulse labeled and the radiolabeled, deglycosylated intermediate of RI_332_ was enriched during a 90 min chase period (Figure 4C). After selective permeabilization of the plasmamembrane by PFO, the cytosolic fraction was separated from cellular remnants by centrifugation, in the presence or absence of added high salt to ensure release of peripherally membrane-associated materials. Only for UBX-EBV WT cells did we see release of the deglycosylated RI_332_ (RI332 – CHO) into the supernatant fraction (S), even more pronounced in the presence of high salt (S'). However, glycosylated RI_332_ (RI332 + CHO) was retained in the pellet fraction under all conditions, consistent with an ER-luminal localization. PFO permeabilization did not damage intracellular compartments, as verified by complete retention of the ER-resident chaperone PDI (Figure 4C) in the particulate fraction.

We conclude that the deglycosylated form of RI_332_ was indeed dislocated from the ER and arrived in the cytosol. Combined, our observations show that expression of UBX-EBV WT uncouples dislocation and degradation of RI_332_. We wondered if the deglycosylated intermediate of RI_332_ once cytosolic would remain associated with the ER or whether it might travel to a different location. Immunofluorescence microscopy showed that RI_332_ localized to the ER in UBX-EBV WT expressing cells, with no evidence of obvious aggregation (Figure 4D). In light of the fractionation data, this suggests that upon dislocation, a sizable fraction of deglycosylated RI_332_ remains loosely associated with the cytosolic face of the ER-membrane.

### A DUB-Catalyzed Reaction Is Essential for Protein Dislocation

We blocked dislocation from the ER by the expression of YOD1 C160S, which causes stabilization of ER resident, glycosylated RI_332_ [11]. We previously proposed that p97-mediated dislocation is stalled under these conditions, because Ub removal is required to allow threading of the dislocation substrate through p97's central pore [11]. If this interpretation is correct, then it should be possible to reverse this block by expression of a DUB capable of attacking the hypothetical stalled intermediate and to overcome the YOD1 C160S-imposed block. Indeed, co-expression of comparable levels of UBX-EBV WT and YOD1 C160S (Figure 4E) resulted in the accumulation of the deglycosylated intermediate of RI_332_ indicative of dislocation (Figure 4F). Co-expression of the inactive mutant UBX-EBV I173W failed to do so, thus excluding simple competition of the UBX-fusion protein with other p97-interactors as the explanation. Consistently, even the non-targeted EBV-DUB without fused UBX-domain could relieve the blockade of dislocation imposed by YOD1 C160S (Figure 4F).

We conclude that a DUB-catalyzed reaction is essential for protein dislocation from the ER. Because ubiquitylation by HRD1-SEL1L is required for the initial engagement of the cytosolic dislocation apparatus [2],[34],[40], premature removal of ubiquitin might also inhibit the earliest steps in this pathway, if EBV-DUB has access to these ubiquitylated intermediates. The rate of dislocation as determined by the disappearance of glycosylated RI_332_ (Figure 3B; RI332 + CHO) was not affected compared to control cells in EBV-WT expressing cells, but was lower in cells expressing the p97-targeted variant (Figure 3B, UBX-fusion; Figure S3). Thus, the non-targeted form of the EBV-DUB interfered exclusively with the degradation of already dislocated RI_332_, while the ER-targeted variant stabilized RI_332_ at the initiation of dislocation and through prevention of delivery to the proteasome by preemptive removal of poly-Ub chains from the substrate. Nevertheless, both variants, p97-targeted or not, caused accumulation of deglycosylated, dislocated RI_332_ in the cytosol.

### Staging a Misfolded ER-Derived Glycoprotein on Its Path to Destruction

What keeps the breakdown intermediate(s) of RI_332_ from aggregation? To address this question and to gain a more global perspective on the natural history of a misfolded protein, we staged the different steps in degradation through identification of proteins that interact with RI_332_ and its various dislocation intermediates. Through interference with dislocation and degradation by different means, we generated discrete intermediates in the breakdown pathway of RI_332_ as explained in the preceding sections. Using affinity tagged RI_332_ as a bait, we retrieved interacting proteins from UBX-EBV WT, YOD1 C160S, or p97 QQ-expressing (an ATPase-deficient form of p97) cells [6], and from cells exposed to the proteasome inhibitor ZL3VS or eeyarestatin I, an inhibitor of dislocation and possibly membrane insertion [29],[41],[42]. As controls, we performed immunoprecipitations from cells that either did not express RI_332_ or that co-expressed RI_332_ with YOD1 ΔZnf C160S, YOD1 WT, or UBX-EBV I173W, none of which significantly perturb the dislocation/degradation process [11]. We performed a total of nine independent large-scale immunopurifications and analyzed each by LC/MS/MS. We identified 836 candidate interactors and enumerated the number of peptides that originated from each one of them. We sought to identify interactions enriched upon inhibition of dislocation/degradation to gain insight into their spatial and temporal occurrence. We therefore normalized our dataset as follows. Each candidate protein was represented by multiple peptide fragments in different experimental conditions, and the maximum number of peptides (MNOP) for a given candidate was based on the condition that yielded the highest peptide count. All interactions between RI_332_ and its candidate interactors (retrieved from independent immunoprecipitation experiments) were expressed as a percentage of the MNOP. Such a normalized interaction matrix should facilitate the identification of groups of proteins that responded similarly if certain discrete steps of the dislocation/degradation pathways are perturbed.

**Figure 5:**
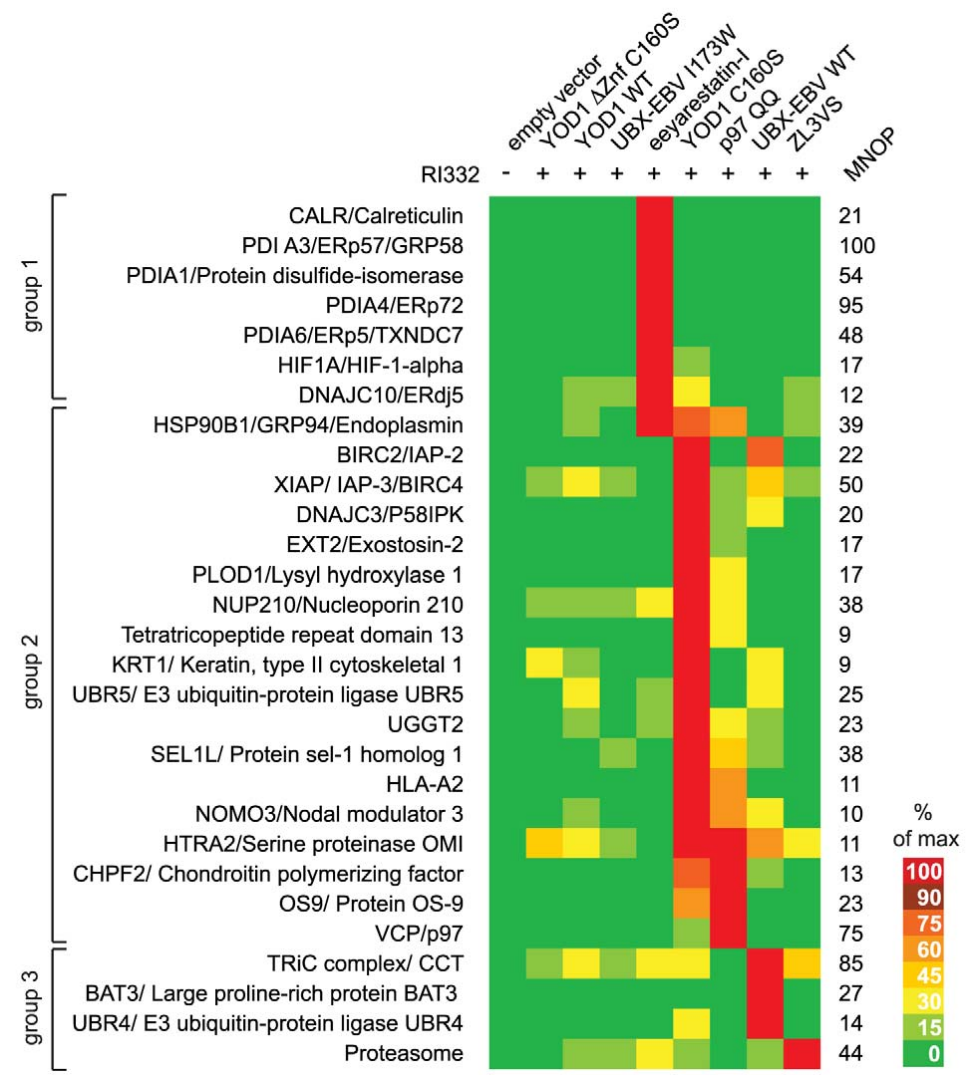
The natural history of an unstable, ER-luminal glycoprotein. RI_332_ was immunoprecipitated from cells transfected with indicated constructs or treated with eeyarestatin-I or ZL3VS (10 µM each). As control, one batch of cells (empty vector) was not transfected with RI_332_. Co-immunoprecipitated interactors of RI_332_ were identified by LC/MS/MS. The maximum number of peptides (MNOP) associated with RI_332_ for a given condition was set to 100% and peptides recovered from the other experimental conditions expressed as percentage of the maximum for each candidate accordingly. After selection using a strict set of rules (see Materials and Methods), the candidates were grouped via K-means clustering. See also the list of candidate interactors in Table S1. The color-coding represents a quantitative measure for the interaction from bright red (maximal interaction) to dark green (minimal/no interaction).

After application of a stringent set of rules (inclusion requirement based on a threshold number of peptides and absence from normal serum controls; see Text S1 for details) a total of 33 candidate interactors remained (Table S1). The candidates were arranged in three groups via k-means clustering and are depicted in a heat map (Figure 5). The heat map is a graphical representation of the normalized interaction matrix and visualizes the conditions under which a particular interactor co-precipitated with RI_332_.

Group 1 comprised those interactors of RI_332_ that were specifically retrieved from eeyarestatin-I treated cells (Figure 5). Consistent with an ER-luminal accumulation of RI_332_, we observe the ER-luminal disulfide shuffling and/or PDI-domain containing proteins (ERdj5, ERp72, ERp57, ERp5, PDI, and Calreticulin) in association with RI_332_ (see Table S1 for Swiss-Prot IDs of candidate interactors) [42],[43].

When a protein is terminally misfolded, a family of substrate recognition molecules targets the substrate to the dislocation machinery. A snapshot of this type of intermediate was provided by co-expression of YOD1 C160S or mutant p97 (QQ). Specifically enriched interaction partners of RI_332_ under these conditions clustered in group 2 and include glycan-binding and modifying proteins (OS9, UGGT2), general ER-luminal substrate recruiting factors and chaperones (SEL1L, Endoplasmin/GRP94, DNAJC3/P58IPK), p97, and the cytosolic UBR5, implicated in ubiquitylation according to the N-end rule [6],[44]–[46]. Our unbiased proteomic approach supports our earlier proposal that YOD1 C160S blocks the dislocation reaction itself [11]. RI_332_ is recognized as misassembled by OS9, SEL1L, and GRP94, associates with p97, but cannot be extracted in YOD1 C160S and p97 QQ cells [11],[34],[40],[45]. Interactors that clustered in group 2 comprised both ER-luminal and cytosolic components of the ER-quality control machinery and UPR signaling.

After successful dislocation, misfolded proteins are targeted to the cytosolic proteasome. Group 3 comprised those proteins that were co-immunoprecipitated with RI_332_ from UBX-EBV WT or ZL3VS treated cells. Our mass spectrometry data suggested that the deglycosylated intermediate of RI_332_ associates with cytosolic chaperones, namely the co-chaperone recruiter BAT3 and the TRiC complex/CCT. The TRiC complex is important for transient stabilization of nascent polypeptide chains prior to their translocation into the ER [47],[48], and BAT3 was recently implicated in integration of tail-anchored proteins into the ER membrane [49]. Not surprisingly, we can demonstrate an interaction of RI_332_ with the proteasome when its activity was blocked by the action of ZL3VS. The identified cytosolic interactors of RI_332_ fully support our biochemical characterization and suggest that dislocation and degradation are uncoupled in UBX-EBV WT expressing cells.

### A Cytosolic Chaperone Associates with an ER-Derived Dislocated Protein

**Figure 6:**
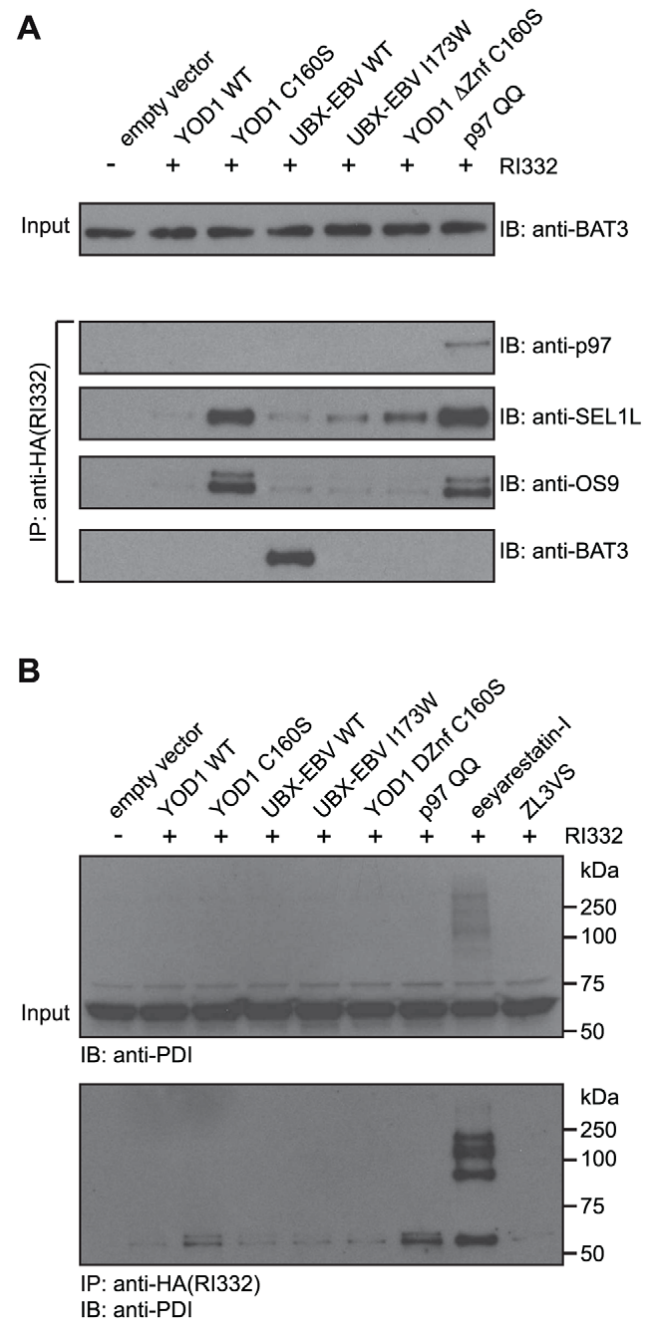
BAT3 interacts with the cytosolic dislocation intermediate of RI_332_ and eeyarstatin-I induces adducts of PDI. (A, B) 293T cells transiently transfected as indicated were homogenized in NP40 lysis buffer and subjected to immunoprecipitation with anti-HA antibodies. As control, one batch of cells was not transfected with RI_332_. Co-precipitated interactors were identified by immunoblotting using the antibodies anti-BAT3, anti-p97, anti-SEL1L, anti-OS9 (left panels), and anti-PDI (right panel). To control for equal loading, lysates were subjected to immunoblotting using (A) anti-BAT3 and (B) anti-PDI antibodies. See also the structural formula of eeyarestatin-I in Figure S6.

As corroboration of the mass spectrometry experiments, we verified interactors of RI_332_ (Figure 6A) by different means. After immunoprecipitation of RI_332_ from cells transfected/treated as in the large-scale pulldown experiments, we confirmed an interaction of RI_332_ with p97 when the ATPase activity of p97 was blocked by mutation (p97 QQ). We likewise confirmed the association of SEL1L and OS9 with RI_332_ when co-expression of either YOD1 C160S or p97 QQ blocked its dislocation from the ER. We established the association of RI_332_ with BAT3, which required the co-expression of UBX-EBV WT. These data hint at the existence of a chaperone-mediated buffer to sequester dislocated proteins from the canonical degradation pathway.

The mass spectrometry experiment predicted a strikingly enriched association of PDI/PDIA1 with RI_332_, when cells were treated with eeyarestatin-I. Indeed, we could verify an interaction of these proteins when co-expressed with either YOD1 C160S or p97 QQ or when cells were treated with eeyarestatin-I (Figure 6B). More surprisingly, eeyarestatin-I induced the formation of SDS- and β-mercaptoethanol-resistant adducts of PDI. The enrichment of these adducts relative to monomeric PDI in immunoprecipitates of RI_332_ was indicative for adduct formation between RI_332_ and PDI upon eeyarestatin-I treatment. Similar observations were also made for PDIA3/ERp57 (unpublished data).

## Discussion

To explore the contributions of ubiquitin addition and removal in protein extraction from the ER, we developed an enzyme-based method to disrupt ubiquitin-dependent protein degradation. We used a biochemical approach to analyze its effect on protein turnover and dislocation from the ER.

### A Novel Tool to Block Ubiquitin-Dependent Proteasomal Degradation

The expression of a highly active viral DUB markedly shifts the cellular balance towards deubiquitylation (Figures 2A, S1). Similar to pharmacological inhibition of the proteasome, accelerated or premature Ub removal from substrates should affect their ubiquitin-dependent degradation. Indeed, overexpression of the EBV-DUB blocks the degradation of several model substrates, but without the immediate cytotoxic effects that are commonly observed upon treatment of cells with pharmacological proteasome inhibitors (Figure 2C,D). In view of Ub's role in cell cycle control, through K_11_-linked Ub-chain assembly by the ubiquitin ligase APC/C [50], the ultimate demise of cells with an arrested UPS is of course hardly surprising. The greater cytotoxicity of pharmacological inhibition could perhaps be related to ubiquitin-independent functions of the proteasome or reflect the critical importance of free Ub in the cell [3],[51]. Interference with the UPS by blocking proteolysis with small molecules or by enzymatic interference with proteasomal targeting are two fundamentally different approaches. Small molecule proteasome inhibitors cause an accumulation of polyubiquitylated proteins and deplete cells of free Ub [52]. Cell viability critically depends on a pool of free Ub and its depletion kills cells [51]. Shifting the cellular balance towards deubiquitylation, as achieved by the EBV-DUB, does not result in accumulation of polyubiquitylated proteins (Figure 2A) and represents a novel means of inhibiting the UPS. Unlike the EBV-DUB, pharmacological inhibition of the proteasome reduces *de novo* protein synthesis even after relatively short times of exposure (Figure 2C) [30],[31]. Translation, protein folding, secretion, and dislocation are interdependent processes and the ability to block proteasomal protein degradation without immediately affecting translation provides an additional benefit.

### Deubiquitylation Is Essential for Protein Dislocation from the ER

**Figure 7:**
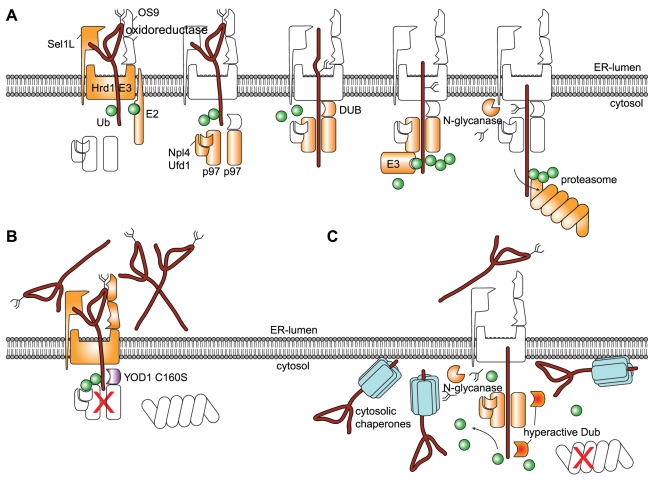
A misfolded glycoprotein on its path to destruction. (A) Model of two consecutive ubiquitylation cycles that initiate dislocation and target the substrate to the proteasome, respectively. (B) Expression of catalytically inactive YOD1 C160S impairs deubiquitylation upstream of the p97 AAA ATPase. This jams the central pore of p97 and processive threading of the dislocation substrate is arrested. In consequence, misfolded, glycosylated proteins accumulate in the ER-lumen and as partially dislocated intermediates at the site of dislocation. (C) Expression of the highly active EBV-DUB deubiquitylates misfolded proteins upstream of p97 and thus facilitates processive dislocation mediated by p97. Lack of proper proteasome commitment results in accumulation of a deglycosylated, cytosolic intermediate of the misfolded protein.

We showed previously that YOD1 C160S causes complete retention of RI_332_ in the ER, a substrate otherwise extracted and targeted for degradation (Figure 7A,B) [11]. This retention can be reversed at least in part by expression of the p97-targeted EBV-DUB (Figures 4F, 7C). Combined, these results demonstrate a need for removal of Ub to achieve dislocation. Ramping down the p97-associated DUB activity blocks dislocation but can be rescued by an active DUB. This immediately suggests that DUBs can have opposing functions for the degradation of ER-derived proteins. Some DUBs might impair dislocation by reversing ubiquitylation; others might facilitate dislocation and subsequent degradation. Indeed, TCRα-GFP is stabilized by knockdown of USP13 but destabilized by knockdown of Ataxin-3 [12]. Moreover, the observed uncoupling of dislocation and degradation by the EBV-DUB suggests that a persistently ubiquitylated state is not essential for the physical extraction of substrate from the ER. Misfolded proteins are escorted to the proteasome by ubiquitin binding proteins [53],[54]. It is therefore no surprise that the removal of Ub-chains by the EBV-DUB abrogates proteasomal turnover of these proteins. By analogy, p97-mediated extraction would be arrested by the EBV-DUB to a similar extent if persistent substrate modification with Ub-chains were required to exert a mechanical force for such extraction. Indeed, protein dislocation is slowed down, but clearly the reaction continues in cells that express EBV-DUB (Figures 4A, S3). Together with the evident requirement of a DUB-catalyzed reaction upstream of p97 (Figure 4E), these findings suggest that Ub-chains are not required to exert a mechanical force on the dislocation substrate [7] but may instead be required only as recognition signal [5].

Our data are consistent with the following model (Figure 7A). Dislocation substrates are targeted to a dislocon that connects with an Ub ligase activity, as exemplified by the HRD1-SEL1L complex [2],[34],[45]. Consistent with its requirement for dislocation, substrate ubiquitylation recruits p97 and the Ub-recognizing co-factors Ufd1 and Npl4 to initiate dislocation [6],[7],[11],[12],[55]. Once the dislocation machinery is recruited, one or several p97-associated DUBs remove the initial Ub tag to enable ATP-dependent threading through the central pore of p97 [7],[11],[12],[55]. Unless p97 is coupled directly to the proteasome, for which there is no firm experimental support at present [56], a second round of ubiquitylation would be required to target the unfolded protein to the proteasome, again necessitating removal of the Ub-chain prior to its insertion into the proteolytic chamber [14]. There is of course also a structural resemblance between the 6-fold symmetrical p97 complex, present in association with Ub-recognizing and -processing factors, and the similarly equipped proteasomal cap complex [53].

The first round of ubiquitylation would be responsible for the engagement and proper assembly of the p97-Ufd1-Npl4 dislocation complex and may involve K_11_-linked ubiquitin chains [21],[57]. The second round would then target the dislocated, now cytosolic protein to the proteasome like any other p97-dependent, cytosolic substrate of the UPS [12],[29],[57]. Ubiquitylation-dependent events occur both upstream and downstream of p97 [53],[54]. Two consecutive rounds of ubiquitylation are conceptually similar to the use of multiple independent ubiquitylation sites on many standard proteasomal substrates: modification of more than a single site on a given substrate requires sequential engagement by a given ligase, or the involvement of more than one ligase.

The p97-targeted EBV-DUB interferes with degradation of ER-derived substrates at two distinct steps (Figure 7C). First, it interferes with proper initiation of dislocation by premature removal of ubiquitin. Second, it blocks substrate degradation by removal of the poly-Ub chains that would otherwise have mediated delivery of the misfolded protein to the proteasome. In the absence of a fused UBX-domain, the EBV-DUB affects to a lesser extent the initial stages of dislocation but still strongly inhibits proteasomal proteolysis of cytosolic substrates and ER-derived substrates (Figures 2B, 3A,B). The EBV-DUB can even facilitate the dislocation reaction when dislocation is otherwise stalled by expression of YOD1 C160S (Figures 4F, 7B).

Our data establish the necessity of a DUB-catalyzed reaction upstream of p97-mediated protein extraction from the ER. These observations are most consistent with a model of two consecutive rounds of ubiquitylation and deubiquitylation.

### The Natural History of an ER-Luminal Glycoprotein Dislocation Substrate

Using different tools to block protein dislocation and degradation, we identified interacting partners of a misfolded, ER-derived glycoprotein at different stations on its road to destruction. Starting in the ER, eeyarestatin-I was first identified as an inhibitor of dislocation [42]. However, there is no consensus on the identity of its molecular targets or its exact mode of action [29],[41],[58]. Eeyarestatin-I contains two halogenated benzene rings (Figure S6). Bromobenzene, a hepatotoxic compound, is metabolized to reactive metabolites (e.g. bromobenzene-3,4-oxide) and forms covalent adducts with cellular proteins, including PDIA1, PDIA6, and PDIA3 [59],[60]. Also other halogenated aromatic derivatives covalently modify proteins in a reaction mechanism similar to that involving bromobenzene [61]. If the two halogenated benzene rings of eeyarestatin-I would react similarly, formation of covalently cross-linked adducts of PDI might ensue, consistent with our observations that eeyarestatin-I induces SDS-resistant adducts of PDI (Figure 6B). We therefore suggest the possibility that the two halogenated benzene rings of eeyarestatin-I might promote crosslinking of proteins in the ER lumen and that its targets include the machinery for disulfide shuffling. If the machinery for disulfide shuffling in the ER is indeed a molecular target of eeyarestatin-I, then both import into and export from the ER are likely to be affected, explaining the seemingly divergent observations reported for eeyarestatin-I's mechanism of action [29],[41],[42].

By staging the process of dislocation and degradation we identified several novel and intriguing candidate interactors for the RI_332_ dislocation substrate, including proteins important in the maturation of extracellular matrix components (PLOD1, CHPF2, EXT2). Whether the machineries for heparan sulfate synthesis, chondroitin sulfate synthesis, and collagen polymerization merely undergo rapid turnover and are processed via p97 or whether they are otherwise involved in the dislocation reaction remains to be established.

We validated BAT3 as a cytosolic interactor of the deglycosylated intermediate of RI_332_. This result implies not only that RI_332_ is dislocated from the ER but also that it remains associated with chaperones, possibly to prevent aggregation. This type of interaction may be an example of how cells cope with dislocated proteins that escape degradation. Could it be that the machineries for translocation and dislocation share certain co-factors? Intriguingly, BAT3 has recently been implicated in proteasomal degradation of newly synthesized defective polypeptides [62]. This might suggest that cytosolic quality control machineries handle defective, ribosome-derived nascent chains similarly to how they deal with defective polypeptides that originate from the ER. The specifically enriched association of the TRiC/CCT with RI_332_ in UBX-EBV WT expressing cells (Figure 5) further supports this interpretation: Cytosolic chaperone complexes implicated in the stabilization and quality control of folding intermediates prior to translocation in the ER [47],[48] also interact with an ER-derived, dislocated protein.

The approach developed here—expression of the EBV-DUB—stabilizes a range of proteins that are normally degraded in an Ub-dependent manner. Although capable of blocking Ub-dependent protein degradation globally and efficiently, the EBV-DUB is less toxic to cells than pharmacological proteasome inhibitors, providing an extended window of observation. We show that dislocation and degradation of ER-derived misfolded substrates can be uncoupled by the expression of the viral DUB domain and so allows the unprecedented visualization of a deglycosylated dislocation intermediate in the absence of pharmacological proteasome inhibitors. We identify the necessity of a DUB-catalyzed reaction for protein dislocation from the ER and place this activity upstream of p97. The determination of the sets of interacting partners of a misfolded, ER-derived glycoprotein at different stations on its road to destruction helped us characterize the order of events during dislocation and degradation. The expression of this highly active DUB has provided new mechanistic insights into protein quality control. Given its low toxicity and the possibility of achieving cell-type or tissue-specific expression in vivo, the EBV-DUB and variants derived from it may prove to be an attractive alternative to the use of small molecule inhibitors of the proteasome.

## Materials and Methods

### Antibodies, Cell Lines, Constructs, Experimental Procedures

Antibodies against the HA-epitope were purchased from Roche (3F10); anti-Flag, anti-Ubiquitin antibodies were purchased from Sigma-Aldrich; and Lys48-specific anti-Ubiquitin antibodies (anti-K48-Ub, clone Apu2) were purchased form Millipore. Anti-p97 and anti-BAT3 antibodies were purchased from Fitzgerald Industries and Abcam, respectively. Polyclonal anti-OS9 and anti-SEL1L antibodies were described previously [45],[63]. A.E. Johnson (Texas A&M University, TX) provided a plasmid encoding perfringolysin O. Polyclomal anti-PDI serum (rabbit) was generated with bacterially expressed human PDI. 293T cells were cultured and transfected as previously described [63]. The deletion constructs and mutants of YOD1 have been described elsewhere [11]. All p97-targetting constructs were cloned into the pcDNA3.1(+) vector system (Invitrogen) with a Kozak sequence (GCCACC) inserted directly upstream to the Start-Codon, and encoded for a N-terminal Flag-tag (DYKDDDK) followed by the UBX domain of YOD1 (aa1–131). For the UBX-GFP construct, these aa1–131 of YOD1 were followed by a LEGS linker sequence and the aa2–239 of enhanced GFP (EGFP). The UBX-EBV-DUB fusion construct comprised the aa1–128 of YOD1, a GGGS linker sequence and the DUB domain of the EBV large tegument protein BPLF1 (aa1–270). The construct coding catalytically impaired p97 (p97 QQ) was described earlier [11]. Site directed mutagenesis of the EBV-DUB was performed with the QuikChange II mutagenesis kit (Stratagene). The predicted catalytic cysteine residue at position 61 of the original EBV protein BPLF1 was mutated to alanine (C61A), threonine (C61T), serine (C61S), or lysine (C61K). The isoleucine at position 173 and alanine at position 178 were mutated to tryptophane (I173W) and to arginine (A178R), respectively. Maria Masucci provided the Ub-R-GFP and the Ub-G76V-GFP construct. Untagged RI_332_ was a generous gift from N. Erwin Ivessa. The Plasmid pCMV-INSIG-1-Myc was obtained from the American Type Culture Collection (ATCC number 88099). HA-RI_332_ was cloned into pcDNA3.1(+) via *HindIII* and *XbaI* restriction sites. The HA-epitope (YPYDVPDYA) and a GSLE linker sequence were inserted between aa27 and aa28 of the signal sequence.

### Pulse-Chase Experiments, Immunoprecipitations, Gel Electrophoresis, Immunoblotting, and Transient Transfections

Pulse chase experiments were performed as previously described [38]. Prior to pulse labeling, the cells were starved for 30 min in methionine/cysteine-free DMEM at 37°C. Cells were then labeled for 10 min at 37°C with 250 µCi of [^35^S]methionine/cysteine (PerkinElmer). De novo protein synthesis was quantitated in a pulse labeling experiment. Where indicated, 50 µM ZL3VS was applied to the cells during the starvation, pulse labeling, and chase period. Incorporated radioactivity was quantified after SDS-mediated cell lysis and TCA precipitation.

Transient transfection, cell lysis, immunoprecipitations and transfections, SDS-PAGE, and fluorography were performed as described earlier [34]. All quantifications were performed on a phosphoimager.

For the protease protection assay cells were homogenized by passing through a 23 x g needle in hypotonic buffer (20 mM Hepes pH 7.5, 5 mM KCl, 5 mM MgCl_2_, 1 mM DTT, and a protease protection cocktail (Roche)). Proteinase K was added to a final concentration of 100 µg/ml in the presence and absence of 0.5% NP40. After 20 min on ice, the proteinase K was inactivated by inclusion of PMSF (5 mM) and samples were analyzed by SDS-PAGE.

### Selective Plasmamembrane Permeabilization and Subcellular Fractionation

For selective permeabilization of the plasmamembrane, cell 293T cells were trypsinized harvested and washed with PBS. Perfringolysin O was added to a final concentration of 0.5 µM at 0°C followed by an incubation of the cells at 37°C for 15 min to induce pore-formation. Where indicated, the mixture was adjusted to 0.5 M NaCl. Centrifugation (5 min, 9000 x g) separated cytosolic proteins in the supernatant from cellular remnants in the pellet. The pellet was washed with ice-cold PBS and lysed in PBS/1% SDS to solubilize membrane proteins and release organelle contents.

### Structural Modeling

Structural modeling is described in Text S1.

### Data Analysis and Clustering

Data analysis and clustering are described in the Supporting Information section.

### Confocal Microscopy

Cells were grown on coverslips, fixed in 4% paraformaldehyde, quenched with 20 mM glycerine, 50 mM NH_4_Cl, and permeabilized in 0.1% Triton X-100 at room temperature. Fixed and permeabilized cells were blocked in 4% BSA and incubated either with anti-HA antibodies (3F10, rat monoclonal, Roche) or anti-PDI (Abcam) antibodies as described [45]. Images were acquired by using a spinning disk confocal microscope, a Nikon 60× magnification, and a 1× numerical aperture oil lens.

### Flow Cytometry

Cells were harvested by trypsinization 10 h after addition of proteasome inhibitors and 20 h after transient transfection. Cells were washed and incubated for 30 min at 4°C with a LIFE/DEAD cell viability stain (Invitrogen). Subsequently, cells were washed and fixed with 2% paraformaldehyde. Fluorescence intensity of GFP was measured with LSR I flow cytometer (BD Biosciences). Data were collected with CellQuest (BD Biosciences) and analyzed with FlowJo (Tree Star).

### Cell Morphology

Cells were transiently transfected with EBV-DUB WT or empty vector. After 6 h, pharmacological proteasome inhibitors (10 µM MG132 or 10 µM ZL3VS) were applied to the cells. Photographs were taken 20 h post-treatment.

## Supporting Information

Figure S1 EBV-DUB switches the cellular ubiquitylation balance towards deubiquitylation. 293T cells were transfected as indicated and immunoblotted with anti-Ub (left panel) and anti-K_48_-Ub antibodies (right panel). An immunoblot with anti-BAT3 antibodies serves as loading control. Where indicated, cells were treated for 1 h with 50 µM ZL3VS.
(0.69 MB TIF)

Figure S2 EBV-DUB blocks proteasomal degradation of Ub-G76V-GFP and Ub-R-GFP. Flow-cytometric analysis of 293T cells treated and co-transfected as indicated. The gate was set to identify GFP-positive, live cells. The fraction of GFP-positive cells is given for each panel. Quantified is the median fluorescence intensity (MFI) of GFP-positive cells. Where indicated, the cells were treated with ZL3VS for 10 h prior to formaldehyde-fixation.
(0.52 MB TIF)

Figure S3 EBV-DUB uncouples dislocation and degradation. 293T cells were co-transfected with RI_332_ and UBX-EBV WT and subjected to a pulse chase experiment. After indicated time points, RI_332_ was immunoprecipitated, subjected to SDS-PAGE, and quantified. The fraction of deglycolated RI_332_ (RI332 -CHO) was determined and plotted versus the chase time (right panel).
(0.27 MB TIF)

Figure S4 EBV-DUB can be targeted to p97 by N-terminal attachment of an UBX domain. (A) 293T cells were transiently transfected with the indicated constructs and homogenized in NP40-containing lysis buffer 24 h after transfection. To control for expression of YOD1 variants and chimeric fusions of the YOD1 UBX-domain to GFP and the EBV-DUB, the lysates were subjected to immunoblotting with anti-FLAG antibodies. Anti-p97 antibodies were used to control for equal loading (two upper panels). Retrieved p97 and Derlin-1 from anti-FLAG immunoprecipitates was detected by immunoblotting with anti-p97 and anti-Derlin-1 antibodies, respectively (lower panels). (B) 293T cells were transiently transfected with the indicated constructs. Cell lysates were prepared as in (A). Retrieved p97 and Derlin-1 in anti-FLAG immunoprecipitates was detected by immunoblotting with anti-p97 and anti-Derlin-1 antibodies, respectively (lower panels). The numbers on the left of individual figures represent the molecular weight standard in kDa.
(1.09 MB TIF)

Figure S5 EBV-DUB and the p97-targeted UBX-EBV-DUB block degradation of cytosolic substrates. Flow-cytometric analysis of 293T cells treated and co-transfected as indicated. The gate was set to identify GFP-positive, live cells. The fraction of GFP-positive cells is given for each panel. Quantified is the median fluorescence intensity (MFI) of GFP-positive cells.
(0.30 MB TIF)

Figure S6 Structural formulas of eeyarestatin-I and bromobenzene. The two halogenated benzene rings of eeyarestatin-I are highlighted with red ellipses.
(0.12 MB DOC)

Table S1 Interactome of RI_332_. List of proteins exhibiting enriched interaction with RI_332_ when protein degradation and/or dislocation were blocked by different means (listed in Figure 4). The table gives the protein/gene names, Swiss-Prot accession number, the number of peptides that could be identified under optimal conditions in an LC/MS/MS experiment, the conditions under which the MNOP was observed, and the resulting sequence coverage.
(0.09 MB DOC)

Text S1 Supporting materials and methods.
(0.06 MB DOC)

## Abbreviations

DUB: deubiquitylating enzyme
EBV: Epstein-Barr virus
ER: endoplasmic reticulum
GFP: green fluorescent protein
MFI: median fluorescence intensity
MNOP: maximum number of (co-precipitated) peptides
PAGE: polyacrylamide gel electrophoresis
PBS: phosphate buffered saline
poly-Ub: polyubiquitin
SDS: sodium dodecyl sulfate
TCRα: alpha chain of the T-cell receptor
Ub: ubiquitin
UPS: ubiquitin-proteasome-system
VME: vinyl methyl ester
UFD: ubiquitin fusion degradation
