# Supplementary figures and images for: Enzymatic Blockade of the Ubiquitin-Proteasome Pathway
Source: PLoS Biol. 2011 Mar 29;9(3):e1000605. doi: 10.1371/journal.pbio.1000605 (PMC3066133; doi:10.1371/journal.pbio.1000605)

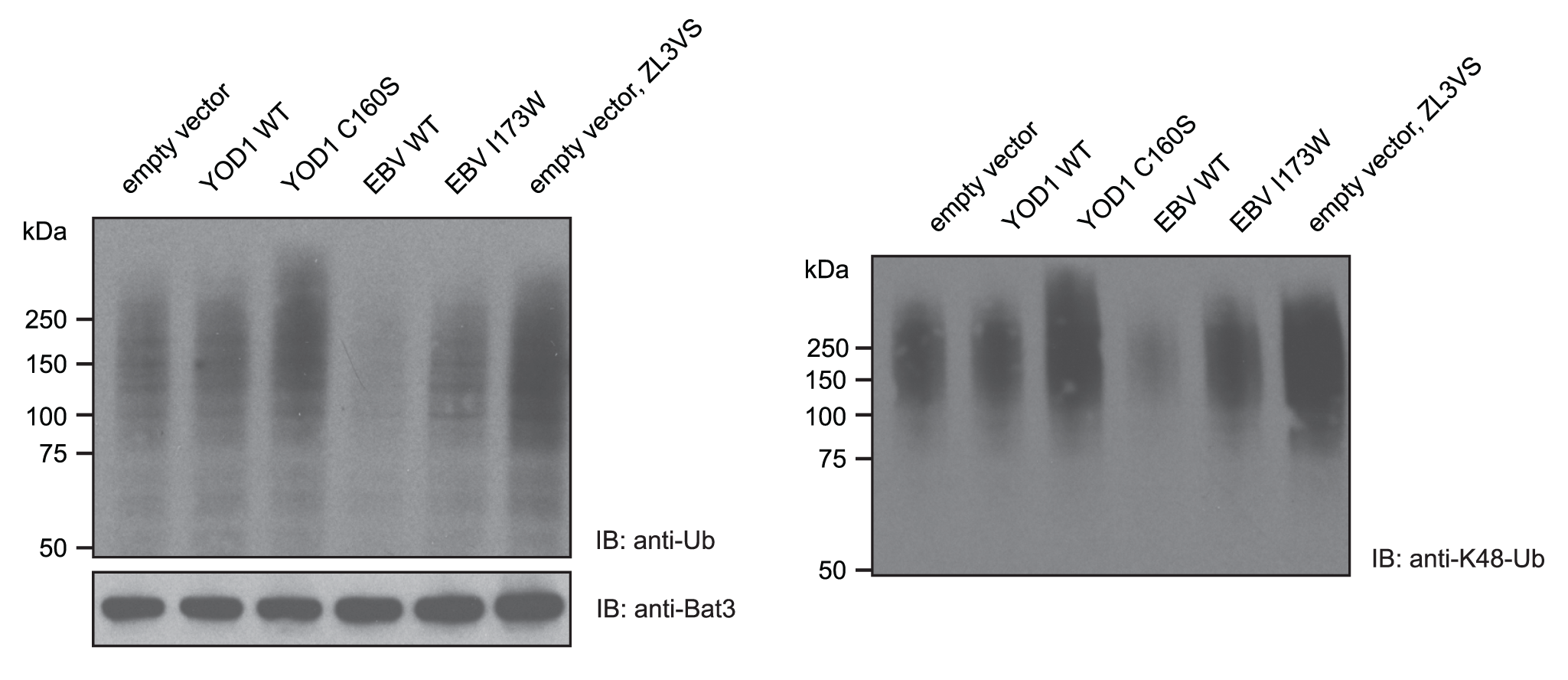

Supplement: Figure S1 — EBV-DUB switches the cellular ubiquitylation balance towards deubiquitylation. 293T cells were transfected as indicated and immunoblotted with anti-Ub (left panel) and anti-K48-Ub antibodies (right panel). An immunoblot with anti-BAT3 antibodies serves as loading control. Where indicated, cells were treated for 1 h with 50 µM ZL3VS. (0.69 MB TIF) [file pbio.1000605.s001.tif]

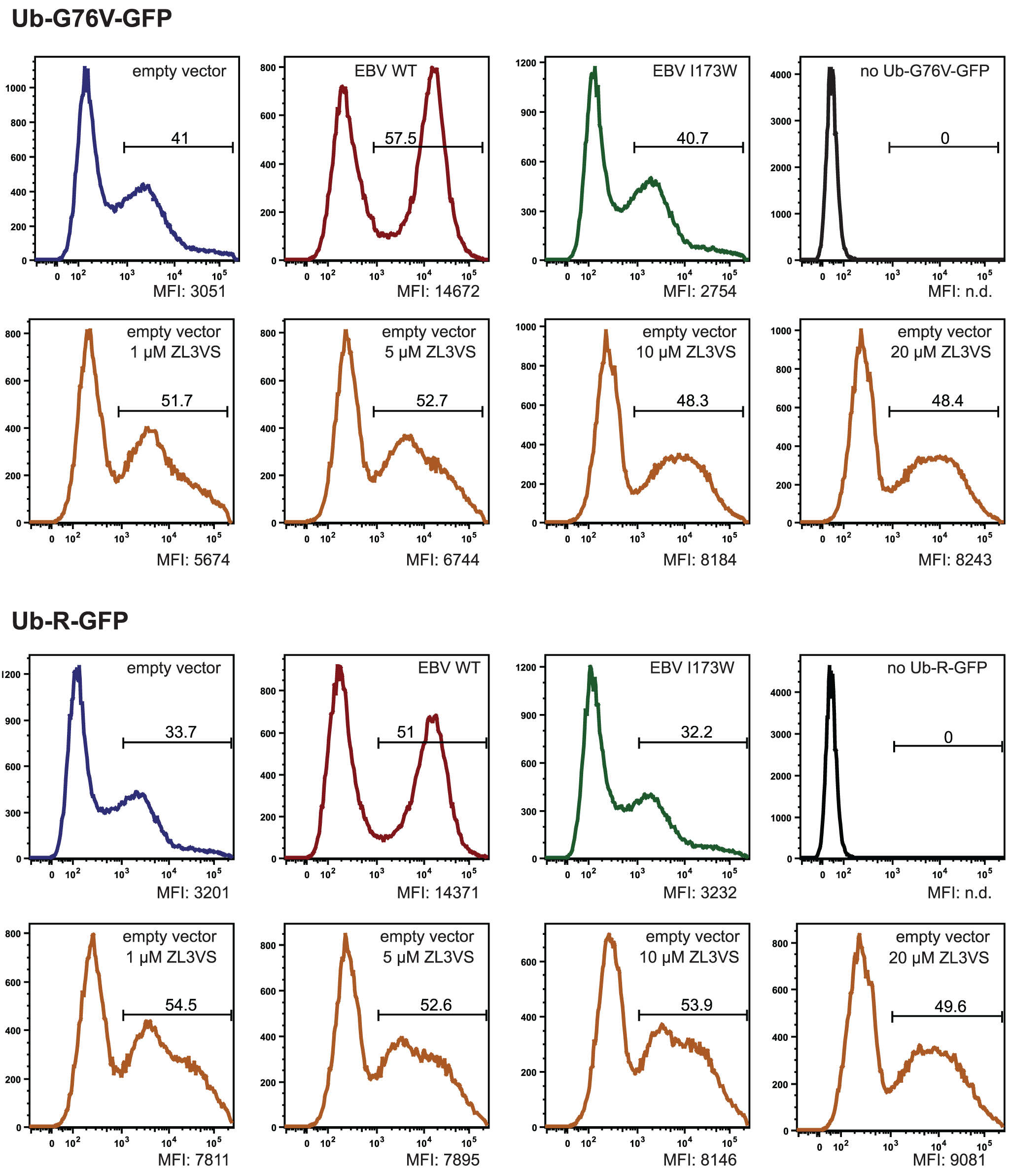

Supplement: Figure S2 — EBV-DUB blocks proteasomal degradation of Ub-G76V-GFP and Ub-R-GFP. Flow-cytometric analysis of 293T cells treated and co-transfected as indicated. The gate was set to identify GFP-positive, live cells. The fraction of GFP-positive cells is given for each panel. Quantified is the median fluorescence intensity (MFI) of GFP-positive cells. Where indicated, the cells were treated with ZL3VS for 10 h prior to formaldehyde-fixation. (0.52 MB TIF) [file pbio.1000605.s002.tif]

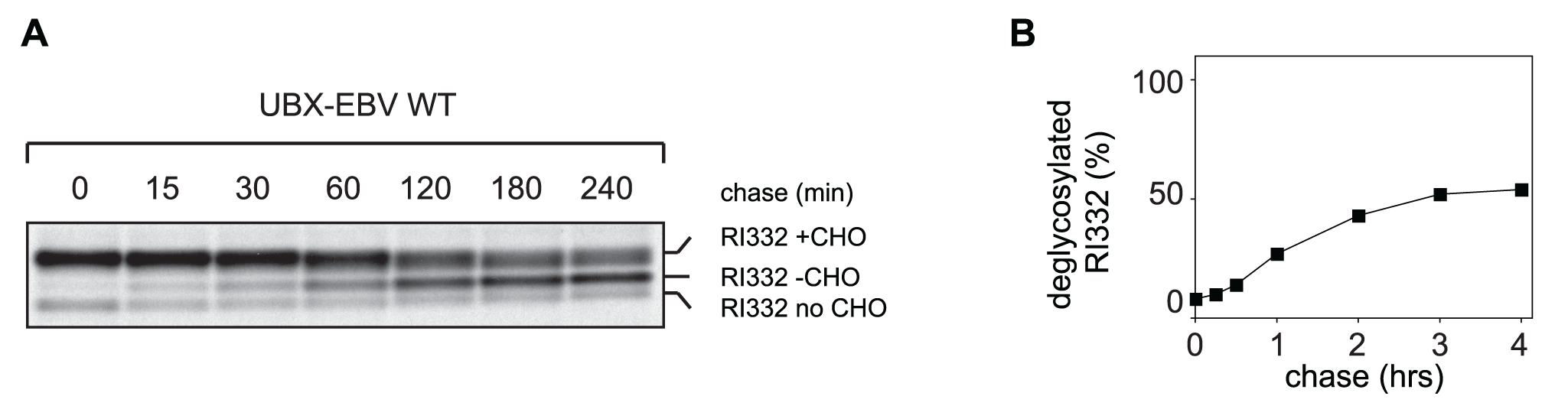

Supplement: Figure S3 — EBV-DUB uncouples dislocation and degradation. 293T cells were co-transfected with RI332 and UBX-EBV WT and subjected to a pulse chase experiment. After indicated time points, RI332 was immunoprecipitated, subjected to SDS-PAGE, and quantified. The fraction of deglycolated RI332 (RI332 -CHO) was determined and plotted versus the chase time (right panel). (0.27 MB TIF) [file pbio.1000605.s003.tif]

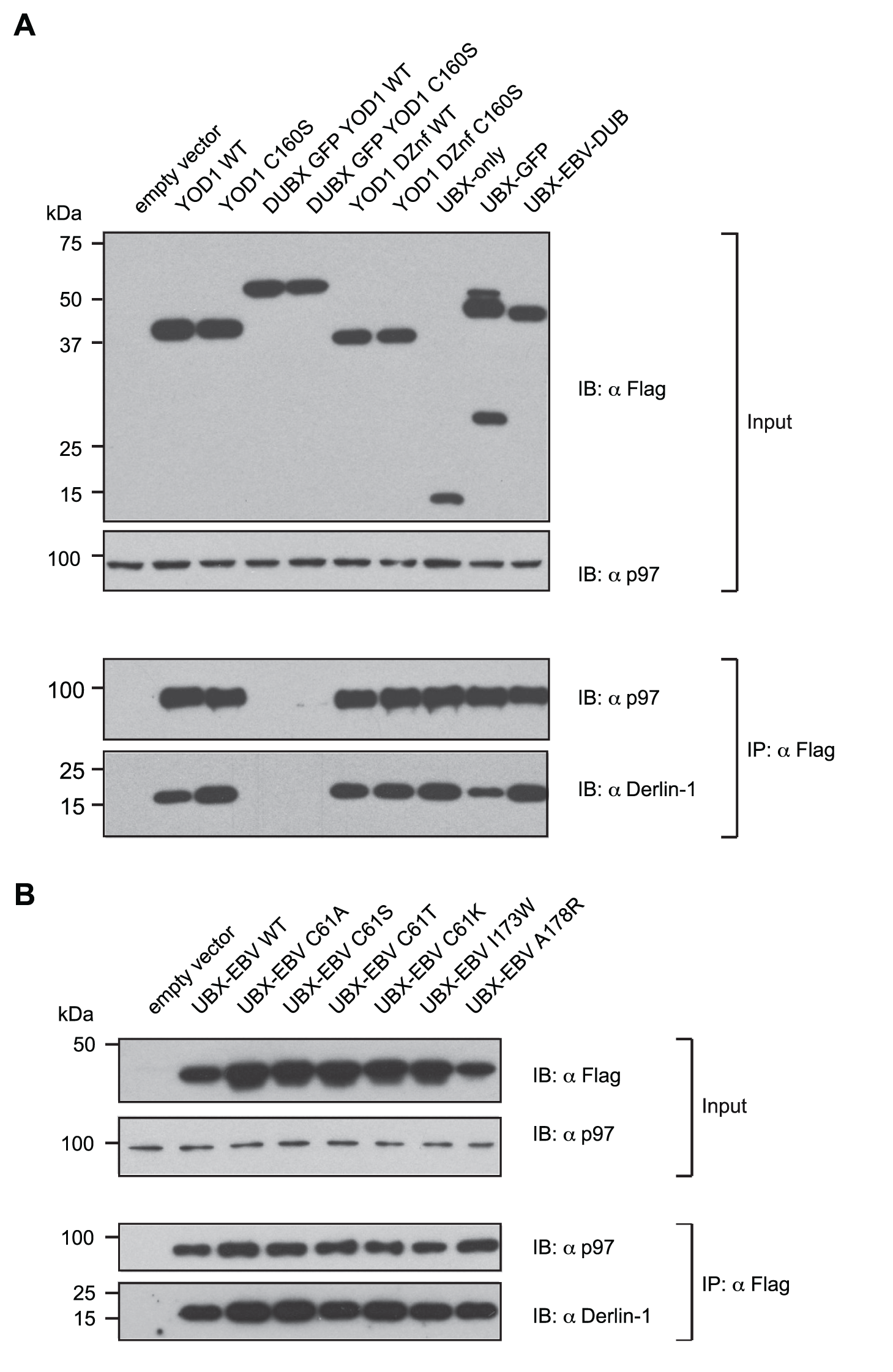

Supplement: Figure S4 — EBV-DUB can be targeted to p97 by N-terminal attachment of an UBX domain. (A) 293T cells were transiently transfected with the indicated constructs and homogenized in NP40-containing lysis buffer 24 h after transfection. To control for expression of YOD1 variants and chimeric fusions of the YOD1 UBX-domain to GFP and the EBV-DUB, the lysates were subjected to immunoblotting with anti-FLAG antibodies. Anti-p97 antibodies were used to control for equal loading (two upper panels). Retrieved p97 and Derlin-1 from anti-FLAG immunoprecipitates was detected by immunoblotting with anti-p97 and anti-Derlin-1 antibodies, respectively (lower panels). (B) 293T cells were transiently transfected with the indicated constructs. Cell lysates were prepared as in (A). Retrieved p97 and Derlin-1 in anti-FLAG immunoprecipitates was detected by immunoblotting with anti-p97 and anti-Derlin-1 antibodies, respectively (lower panels). The numbers on the left of individual figures represent the molecular weight standard in kDa. (1.09 MB TIF) [file pbio.1000605.s004.tif]

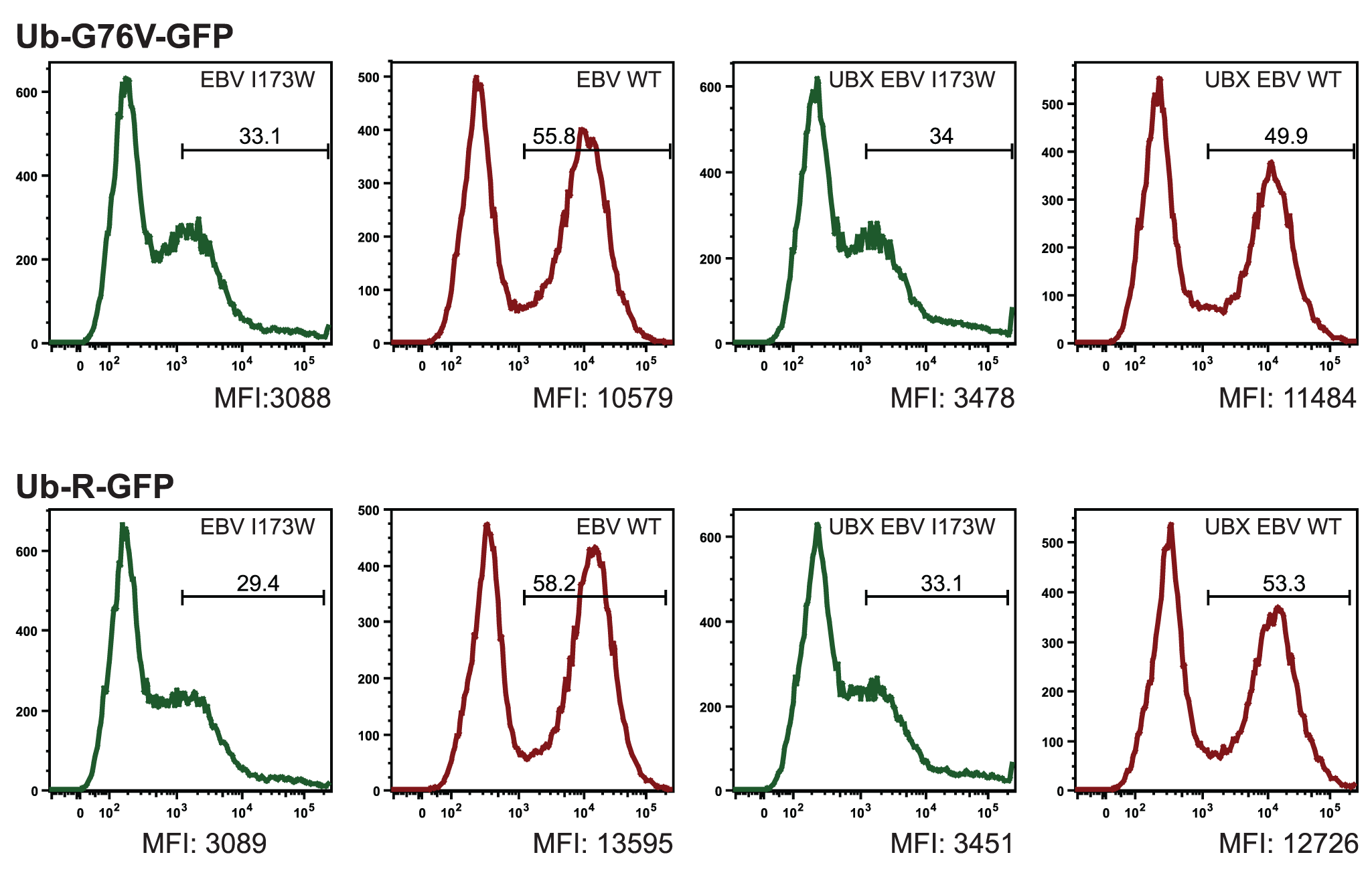

Supplement: Figure S5 — EBV-DUB and the p97-targeted UBX-EBV-DUB block degradation of cytosolic substrates. Flow-cytometric analysis of 293T cells treated and co-transfected as indicated. The gate was set to identify GFP-positive, live cells. The fraction of GFP-positive cells is given for each panel. Quantified is the median fluorescence intensity (MFI) of GFP-positive cells. (0.30 MB TIF) [file pbio.1000605.s005.tif]

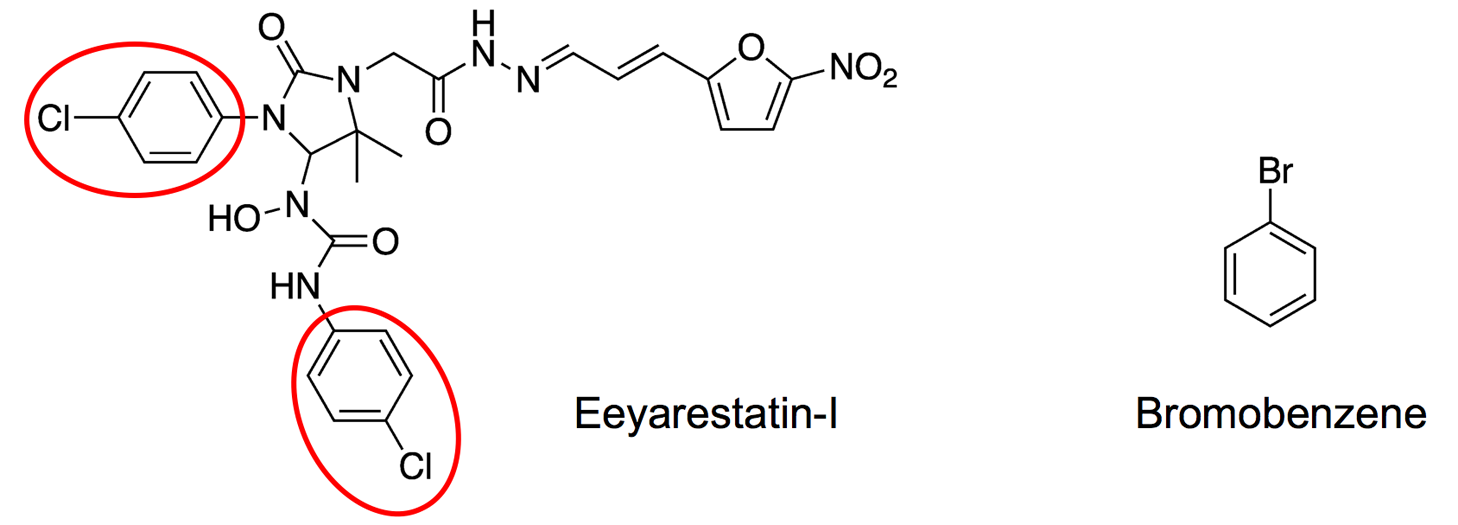

Supplement: Figure S6 — Structural formulas of eeyarestatin-I and bromobenzene. The two halogenated benzene rings of eeyarestatin-I are highlighted with red ellipses. (0.12 MB DOC) [file pbio.1000605.s006.tif]
