# Supplementary material for: Enzymatic Blockade of the Ubiquitin-Proteasome Pathway
Source: PLoS Biol. 2011 Mar 29;9(3):e1000605. doi: 10.1371/journal.pbio.1000605 (PMC3066133; doi:10.1371/journal.pbio.1000605)
Supplement: Table S1 — Interactome of RI332. List of proteins exhibiting enriched interaction with RI332 when protein degradation and/or dislocation were blocked by different means (listed in Figure 4). The table gives the protein/gene names, Swiss-Prot accession number, the number of peptides that could be identified under optimal conditions in an LC/MS/MS experiment, the conditions under which the MNOP was observed, and the resulting sequence coverage. (0.09 MB DOC) [file pbio.1000605.s007.doc]

| **Name** | **Swiss-Prot** | **MNOP / % Coverage** | **MNOP taken from** | **Function** | **Local.** |
| --- | --- | --- | --- | --- | --- |
| IAP-2 | **Q13490** | 22/31 | YOD1 C160S | E3/Apo | Cyt |
| XIAP | **P98170** | 50/66 | YOD1 C160S | E3/Apo | Cyt |
| BAT3/ | **P46379** | 27/23 | UBX EBV WT | F/QC | Cyt |
| CALR/ Calreticulin | **P27797** | 21/25 | Eeyarestatin-I | F/QC | ER |
| TCP1, subunit 2 | **P78371** | 16/28 | UBX EBV WT | F/QC | Cyt |
| TCP1, subunit 3 | **P49368** | 15/28 | UBX EBV WT | F/QC | Cyt |
| TCP1, subunit 4 | **P50991** | 15/23 | UBX EBV WT | F/QC | Cyt |
| TCP1, subunit 5 | **P48643** | 10/19 | UBX EBV WT | F/QC | Cyt |
| TCP1, subunit 8 | **P50990** | 19/38 | UBX EBV WT | F/QC | Cyt |
| CHPF2/ Chondroitin polymerizing factor | **Q9P2E5** | 13/20 | P97QQ | ECM |  |
| DNAJC10/ ERdj5 | **Q8IXB1** | 12/11 | Eeyarestatin-I | F/QC | ER |
| DNAJC3/ P58IPK | **Q13217** | 20/41 | YOD1 C160S | F/QC | ER |
| EXT2/ Exostosin-2 | **Q93063** | 17/29 | YOD1 C160S | ECM |  |
| HIF1A/ HIF-1-alpha | **Q16665** | 17/19 | Eeyarestatin-I | SIG | Cyt |
| HLA-A2 | **P01892** | 11/22 | YOD1 C160S | AP | ER |
| HSP90B1/ GRP94/ Endoplasmin | **P14625** | 39/34 | YOD1 C160S | QC | ER |
| HTRA2/ Serine proteinase OMI | **O43464** | 11/12 | P97 QQ / YOD1 C160S | Apo | Cyt |
| KRT1/ Keratin, type II cytoskeletal 1 | **P04264** | 9/13 | YOD1 C160S | IF | Cyt |
| NOMO3/ Nodal modulator 3 | **P69849** | 10/11 | YOD1 C160S | SIG | ER |
| NUP210/ Nucleoporin 210 | **Q8TEM1** | 38/21 | YOD1 C160S | NPC |  |
| OS9/ Protein OS-9 | **Q13438** | 23/24 | P97 QQ | QC | ER |
| PDIA3/ ERp57/ GRP58 | **P30101** | 100/44 | Eeyarestatin-I | F/QC | ER |
| PDIA1/Protein disulfide-isomerase | **P07237** | 54/43 | Eeyarestatin-I | F/QC | ER |
| PDIA4/ ERp72 | **P13667** | 95/41 | Eeyarestatin-I | F/QC | ER |
| PDIA6/ ERp5/ TXNDC7 | **Q15084** | 48/28 | Eeyarestatin-I | F/QC | ER |
| PLOD1/ Lysyl hydroxylase 1 | **Q02809** | 17/28 | YOD1 C160S | ECM | ER |
| Proteasome 26S subunit ATPase 1 | **P62191** | 9/24 | ZL3VS | UPS/QC | Cyt |
| SEL1L/ Protein sel-1 homolog 1 | **Q9UBV2** | 38/36 | YOD1 C160S | QC | ER |
| Tetratricopeptide repeat domain 13 | **Q8NBP0** | 9/13 | YOD1 C160S |  |  |
| E3 ubiquitin-protein ligase UBR4 | **Q5T4S7** | 14/4 | UBX EBV WT | UPS/E3 | Cyt |
| E3 ubiquitin-protein ligase UBR5 | **O95071** | 25/13 | YOD1 C160S | UPS/E3 | Cyt |
| UGGT2 | **Q9NYU1** | 23/16 | YOD1 C160S | QC | ER |
| VCP/p97 26S ATPase subunit 1 | P55072 | 9/24 | P97 QQ | UPS/QC | Cyt |

**Table S1. Interactome of RI_332_.** List of proteins exhibiting enriched interaction with RI_332_ when protein degradation and/or dislocation were blocked by different means (listed in Figure 4). The table gives the protein/gene names, Swiss-Prot accession number, the number of peptides that could be identified under optimal conditions in an LC/MS/MS experiment with the resulting sequence coverage in percent, and the conditions under which the MNOP was observed. If known, the function of the interactor in protein folding (F), quality control (QC), the UPS (UPS), signaling (SIG), Apopotosis (Apo), antigen presentation (AP), extra cellular matrix formation (ECM), the ubiquitin proteasome system (UPS), as ubiqutin ligase (E3) and as intermediate filament (IF) or part of the nuclear pore complex (NPC) is given. A known localization to the cytosol (Cyt) or endoplasmic reticulum (ER) is indicated.
