# Supplementary material for: Enzymatic Blockade of the Ubiquitin-Proteasome Pathway
Source: PLoS Biol. 2011 Mar 29;9(3):e1000605. doi: 10.1371/journal.pbio.1000605 (PMC3066133; doi:10.1371/journal.pbio.1000605)
Supplement: Text S1 — Supporting materials and methods. (0.06 MB DOC) [file pbio.1000605.s008.doc]

**Supporting Material and Methods.**

*Structural Modeling*

Model coordinates of EBV-DUB were generated using the X-ray structure of M48USP as template (pdb code 2J7Q) after aligning the two sequences in ClustalW (23% sequence identity). MODELLER [1] was used for threading and subsequent refinement with molecular dynamics using simulated annealing. Model quality was assessed using QMEAN [2] and WHATCHECK [3], both showing reliable modeling within the enzyme core region. Ub was docked manually to the EBV-DUB and *in silico* mutations assessed for probable interference with Ub binding. Refined models of mutant EBV-DUB were created using the same procedure as for WT EBV-DUB.

*Data analysis and clustering.*

In order to reduce the number of false positive hits we applied a set of stringent rules: 1) All candidates for which only redundant peptides (peptides that could not be assigned to one specific protein) were excluded (3.6 % of total). 2) All non-human candidates (e.g. rabbit immunoglobulin) were excluded (0.7 % of total). 3) Nonspecifically bound proteins retrieved from cells not expressing RI_332_ were excluded (16.7 % of total). 4) Candidates for which less than nine unique peptides could be detected were excluded (64 % of total). 5) Candidates that had only two-fold or less higher peptides counts upon inhibition of dislocation/degradation as compared to unperturbed dislocation/degradation (YOD1 WT, YOD1 ΔZnf C160S, and UBX-EBV I173W cells) were excluded (10.6 % of total). By applying this very stringent set of rules, we reduced the number of candidate interactors from 836 to 33 (**SOM T1**). Several subunits of the TRiC complex/CCT (Chaperonin containing TCP1) and the ATPase subunit 1 of the 26S proteasome were identified in the final list of candidate proteins (**Su T1**). As these proteins are part of known protein complexes, all peptides derived from subunits of these complexes were considered for further analysis. The final list of candidates was arranged according to their normalized interaction matrix into three groups via K-means clustering (similarity metric: euclidean distance) using the Cluster 3.0 software [4].

**References**

1. Sali A, Blundell TL (1993) Comparative protein modelling by satisfaction of spatial restraints. J Mol Biol 234: 779-815.

2. Benkert P, Tosatto SC, Schomburg D (2008) QMEAN: A comprehensive scoring function for model quality assessment. Proteins 71: 261-277.

3. Hooft RW, Vriend G, Sander C, Abola EE (1996) Errors in protein structures. Nature 381: 272.

4. de Hoon MJ, Imoto S, Nolan J, Miyano S (2004) Open source clustering software. Bioinformatics 20: 1453-1454.
